# Scheduling Projects with Multiskill Learning Effect

**DOI:** 10.1155/2014/731081

**Published:** 2014-02-10

**Authors:** Hong Zha, Lianying Zhang

**Affiliations:** College of Management and Economics, Tianjin University, No. 92 Weijin Road, Nankai District, Tianjin 300072, China

## Abstract

We investigate the project scheduling problem with multiskill learning effect. A new model is proposed to deal with the problem, where both autonomous and induced learning are considered. In order to obtain the optimal solution, a genetic algorithm with specific encoding and decoding schemes is introduced. A numerical example is used to illustrate the proposed model. The computational results show that the learning effect cannot be neglected in project scheduling. By means of determining the level of induced learning, the project manager can balance the project makespan with total cost.

## 1. Introduction

Project scheduling problem is a well-known research field in project management [1], while project scheduling with multiskilled resources is an important extension of it. In the extended problem, activities in the projects are assumed to require multiskills simultaneously to be executed. On the other hand, the resources involved (e.g., human resources) are assumed to possess multiskills and perform one of them at a time. As the assumption of it is closer to the real-world situation, the project scheduling problem with multiskills has attracted much attention in recent years [2–5].

Learning effect might be another nonignorable factor in actual scheduling [6, 7], due to the enormous effect on efficiency of operations. Anzanello and Fogliatto [8] were the pioneer introducing the learning effect into the machine scheduling problem. In addition, Biskup [7] classified the learning effect into two different approaches: the position-based learning and sum-of-processing-time based learning. The position-based learning was considered to be a realistic assumption for machine learning, while the sum-of-processing-time was regarded as a suitable hypothesis for human learning. Most of the researches were carried out based on above-mentioned assumptions. Cheng and Wang [9] simplified linear-log model into linear one in machine scheduling with position-based learning effect. Biskup and Simons [10] proposed the concept of induced learning and investigated to find out an optimal level of induced learning in machine scheduling. Wang [11] considered the machine scheduling problem affected by position-based learning as well as deteriorating jobs. Kuo and Yang [12] developed the model for single processor (e.g., human worker) scheduling problem with sum-of-processing-time based learning. Rudek [13] analyzed the single processor total weighted completion time scheduling problem with time-dependent learning effect and constructed a heuristic algorithm to solve it. Moreover, a few researchers discussed the scheduling problem where the two learning approaches coexisted in some situation. Wu and Lee [14] as well as Lu et al. [15] developed different models for the scheduling problem with both learning approaches.

Although the machine (processor) scheduling problems with learning effect have been fully investigated, the studies on project scheduling problems with learning effect have just started. Wu and Sun [16] were the first to develop a model for project scheduling and human resources allocation, but the situation where human resources possessed multiskills was not considered in the model. Janiak and Rudek [17] proposed a single processor (human worker) scheduling model with multiskill learning effect. But in the model the experience formula was oversimplified where the experience of a worker was assumed to be either 0 or 1; besides it could not be applied to project scheduling problem directly. Heimerl and Kolisch [18] formulated a work assignment model for multiskilled human resources, and many affecting factors were considered in the learning equation. But the equation took the form of position-based approach, which was unsuitable for human learning. Furthermore, the induced learning effect was not considered in the above-mentioned three papers. Their models could not assist the manager to determine an optimal level of induced learning.

To fill this gap, we propose a new model for project scheduling with multiskilled human resources, in which the learning formula is improved. In order to solve the proposed model, a genetic algorithm is introduced.

The remainder of the paper is organized as follows.  Section 2 formulates the project scheduling problem considering multiskill learning effects. Genetic algorithm for solving this problem is described in Section 3. Numerical example is illustrated and discussed in Section 4. Finally, Section 5 concludes the paper.

## 2. Problem Formulation

We consider a project including *J* activities. There are precedence relations between them due to technological requirements. The structure of the project can be depicted as a network **G** = (**V**, **E**). The nodes in the network represent activities, and the arcs between them represent precedence relations. Activity 1 is the source node of the network and activity *J* is the sink node. They are both dummy activities. Each activity*j*  (*j* = 2,…, *J* − 1) is performed by multiskilled human workers. Each worker assigned to the activity can only select one of the skills to perform. When the required quantities of each skill are available, the activity can be started. Once it is started, it may take a certain duration and not be preempted.

The efficiency of human workers might be influenced by learning effects during the project implement. Therefore, the actual duration for each activity might be adjusted in project scheduling. There are two kinds of learning, which are autonomous learning and induced learning. The autonomous learning is learning-by-doing, which means higher efficiency can be achieved by repeating similar activities. The induced learning is the proactive investment in technological knowledge, including worker's training, job-related instructions, and handbooks or supervisors. It can facilitate the learning effect so as to shorten the durations. However, extra cost may be also incurred [10]. Hence, the total cost for project may change too.

In this paper, we develop a novel model for the project scheduling problem with multiskilled worker, in which autonomous and induced learning are both considered. It can assist the project manager to determine the start time, the finish time, and resource allocation for each activity. Moreover, combined with the total cost formula, the project manager can balance the project makespan with the total cost. The proposed model is formulated as follows:

(1)  min⁡ T=fJ

(2)  s.t: fj−fi≥dj, i∈Pj

(3)     dj=max⁡k=1,…,K{∑l=1L∑r=rjklsrjklfdlr}

(4)     dlr={dl1,r=1max⁡{(∑n=1r−1dln)αl,dlm},r≥2

(5)     al=log2(1−xl)LRl, 0≤xl≤xlm<1

(6)     ∑j∈Atasjl≤∑k=1Krskl, At={j ∣ fj−dj<t≤fj}

(7)     j=1,…,J, k=1,…,K, l=1,…,L.

In this model, the objective function (1) minimizes the project makespan *T*, where *f*_*J*_ is the finish time of dummy activity *J*. Constraint (2) represents the precedence relations, where *P*_*j*_ denotes the predecessors of activity*j*. The duration *d*_*j*_ in constraint (2) is calculated by formulas (3)–(5).

According to formula (3), *d*_*j*_ depends on the maximum time that all the workers spend in activity *j*. In formula (3), *d*_*lr*_ denotes the time required for a worker to finish the *r*th unit of job *l*. The ordinal number *r*_*jkls*_ means the accumulative units of job *l* that worker *k* have done since he finished the first unit of that job in activity *j*, while *r*_*jkls*_ means the corresponding accumulative units when he finished the last unit of job *l* in activity *j*.

Formula (4) is the sum-of-processing-time based learning function, which is proposed by Kuo and Yang [19]. In formula (4), *d*_*l*1_ denotes the time required for a worker to finish the first unit of job using skill *l*, which is also the normal time for a worker to finish a unit of that job without learning effect. Parameter *d*_*lm*_ is defined as the minimum value of *d*_*lr*_. In addition, *a*_*l*_ denotes the learning index of skill *l*, which reflects the learning speed on skill *l*.

The value of learning index can be calculated according to formula (5), which is proposed by Biskup and Simons [10]. In formula (5), *x*_*l*_ denotes the induced learning level of skill *l*, and *x*_*lm*_ is defined as the maximum value of *x*_*l*_. Parameter LR_*l*_ indicates the autonomous learning ratio of skill *l*. When *x*_*l*_ is equal to 0, it means that there is only autonomous learning effect existing for skill *l*. Otherwise, both autonomous and induced learning effects for skill *l* are considered.

Constraint (6) represents that the quantity of skills required by activities cannot exceed what the workers can provide. Parameter *as*_*jl*_ denotes the quantity of skill *l* required by activity *j*, while 0-1 binary variable *rs*_*jl*_ indicates whether worker *k* possesses skill *l* or not. When *rs*_*jl*_ is equal to 1, worker *k* possesses skill *l* and vice versa.

Constraint (7) limits the range of *j*, *k*, and *l*.

The formula for extra cost incurred by induced learning is given below:

(8) C=∑j=1J ∑k=1K ∑l=1Lcldjkl+∑l=1Lbx  l2l  +cinT.

In formula (9), *c*_*l*_ denotes the salary paid for a worker using skill *l* per day, *b*_*l*_ indicates the cost coefficient of induced learning for skill *l*, and *c*_in_ is the daily indirect cost.

## 3. Genetic Algorithm

Genetic algorithm was proposed by Goldberg and Holland [20], which was inspired by Darwin's theory of evolution. According to the algorithm, the initial population with suitable encoding and decoding can evolve an optimal solution to real-world problem based on the natural principles of natural selection, crossover, and mutation. Due to its excellent performance, Genetic algorithm was widely used for project scheduling problem [21]. In this paper, we applied a genetic algorithm with specific coding and decoding mechanism to solve the above-mentioned formulation. The procedure of the genetic algorithm is described below.

**Step 1 (initial population)**

Genetic algorithm begins by means of generating an initial population randomly. Parameter pop is defined as the number of individuals in the population. In this paper, the individuals are encoded in the form of activity list. It is a permutation vector of activities conforming to precedence relation, which represents a feasible solution for schedule [22].

**Step 2 (fitness computation)**

To compute the fitness values, we decode the individuals with serial scheduling generation scheme [23], maximum flow algorithm [24], and EFT (early finish time) priority rule.

The serial scheduling generation scheme means the activities are selected according to their order in the list and scheduled at their earliest start period when the available resource is abundant. In order to determine whether the flexible resource allocation at one point is feasible or not, we introduce the maximum flow algorithm proposed by Ford and Fulkerson [24].

**Figure 1:**
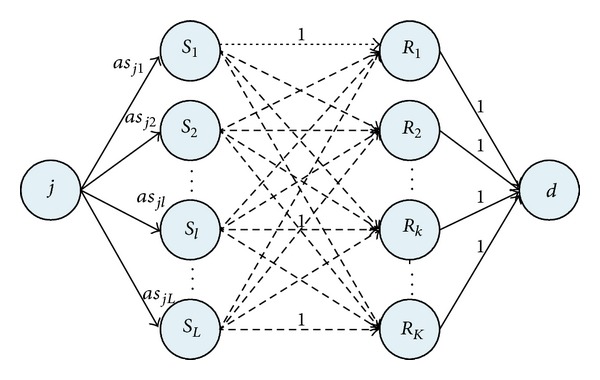
Maximum flow model for flexible resource allocation.

The flexible resource allocation can be modeled as a maximum flow network [25, 26], as shown in Figure 1. In the network, activity node *j* is the source node, while dummy node *d* is the sink node. Between source and sink node, there are skill nodes and resource nodes. The edges connecting activity node and skill nodes reflect the requirement of each skill for activity *j*, where the capacity for each edge represents the required quantity of each skill. While the edges linking resource and skill nodes illustrate the resource-skill relations. According to the relations, the capacity of each edge is set to be either 1 or 0. Besides, the edges connecting resource nodes and dummy nodes show the resource availability at a point in scheduling generation. When the resource is available, the capacity of the edge is set to be 1; otherwise, it is set to be 0. If the maximum flow obtained by the Ford and Fulkerson algorithm is equal to the sum of *as*_*jl*_, the resource allocation for activity *j* is feasible at this moment.

As there may be multiple feasible options for resource allocation of activity *j*, the eventual resource allocation can be determined by EFT priority rule. According to EFT rule, the option with minimized early finish time would be selected.

After decoding, the fitness for individual I can be calculated by the following:

(9) $$\begin{array}{c} \text{fitness}\left(i\right)=\frac{1}{T\left(i\right)}. \end{array}$$

In formula (10), *T*(*i*) denotes the makespan value of individual *i*.

**Step 3 (roulette wheel selection)**

In the selection stage, pop individuals are selected to reproduce according to their fitness values. With roulette wheel method, an individual can be chosen more than once. The selective probability of individual *i* in population can be calculated by the following:

(10) $$\begin{array}{c} p\left(i\right)=\frac{\text{fitness}\left(i\right)}{\sum_{n=1}^{\text{pop}}\text{fitness}\left(n\right)}. \end{array}$$

**Step 4 (partially matched crossover)**

In this step, the partially matched crossover operator is applied on two different individuals from current population with a probability of crossover *p*_cos⁡_.

For two parents *i* ^*f*^ and *i* ^*m*^ selected from the population, after an integer number *q*_cos⁡_  (1 ≤ *q*_cos⁡_ < *J*) is randomly generated, a son *i* ^*s*^ and *i* ^*d*^ are produced. For the son *i* ^*s*^, the first *q*_cos⁡_ activities are taken directly from *i* ^*f*^, while the remaining activities are arranged according to their order in *i* ^*m*^, while the daughter takes the first *q*_cos⁡_ activities from mother and rearranges the remaining according to father.

**Step 5 (swap mutation)**

In this step, the swap mutation operator is applied on newly generated individuals with a probability of mutation *p*_mut_. Swap mutation can be described as follows.

A position *q*_mut_  (1 ≤ *q*_mut_ ≤ *J*) is randomly chosen; then are swapped their contents if activity in position *q*_mut_ + 1 is not an immediate successor of activity in position *q*_mut_. Otherwise, another position is randomly selected. This process repeats until the two adjacent activities can be permuted.

**Step 6 (stop criterion)**

The algorithm stops if the predefined evolutionary generation *gen* is reached. As the stop criterion is satisfied, the optimization result is given as output. Otherwise, the algorithm goes to Step 2 and continues to iterate.

## 4. Computational Experiment

In order to illustrate the model proposed in this paper, a numerical example is given below. As there are no benchmark examples for project scheduling problem with multiskill learning, we randomly generate an example project with multiskilled workers based on the existing research [2]. After that, the learning parameters and cost parameters are added into the generated example. In the example project, there are 12 activities, and the details of them are listed in Table 1. In addition, 10 workers (**w**_1_–**w**_10_) are involved in the project. Each worker is assumed to possess at least one of the four skills (**s**_1_–**s**_4_). The mapping relationships between the workers and the skills are present in Table 2. Besides, the related learning parameters for each skill are shown in Table 3. Finally, the cost parameters in the numerical are given below: *b*_1_ = 2.5 × 10^6^ $, *b*_2_ = 3.5 × 10^6^ $, *b*_3_ = 4.5 × 10^6^ $, *b*_4_ = 3.0 × 10^6^ $, *c*_1_ = 200 $, *c*_2_ = 400 $, *c*_1_ = 500 $, *c*_3_ = 300 $, and *c*_in_ = 1200 $.

In the experiment, the parameters of genetic algorithm are set as follows: population size pop = 50, maximum generation gen = 100, crossover probability *p*_cos⁡_ = 0.95, and mutation probability *p*_mut_ = 0.1.

When the induced learning with top level is considered, the optimal solution is 1-3-2-5-8-6-4-7-10-11-9-12. After decoding it, we obtain a schedule with a makespan of 71.5 days. The optimal schedule is shown in Table 4. In addition, due to the learning effect, workers allocated to the same activity may have various working times. The details on working time in the aforementioned schedule are present in Table 5. From Tables 4 and 5, we can find out start and finish time for activities, resource allocation, and details on working time. For example, we learn that activity 7 is started on day 43.2 and finished on day 58.1. There are 3 workers (**w**_1_–**w**_3_) allocated to the activity. The workesr **w**_1_ and **w**_2_ are assigned to use skill **s**_4_, while worker **w**_1_uses skill **s**_1_. In addition, all of them work in the activity for 14.9 days.

When no learning effect is considered in the project scheduling, the optimal solution is 1-2-4-3-11-7-5-6-8-9-10-12. The corresponding project makespan is 114 days and the optimal schedule is shown in Table 6.

Comparing the two schedules, we find that project makespan can be even shortened by 37.2% based on learning effect. The result demonstrated that learning effect cannot be neglected in project scheduling since it shortens the project makespan.

**Figure 2:**
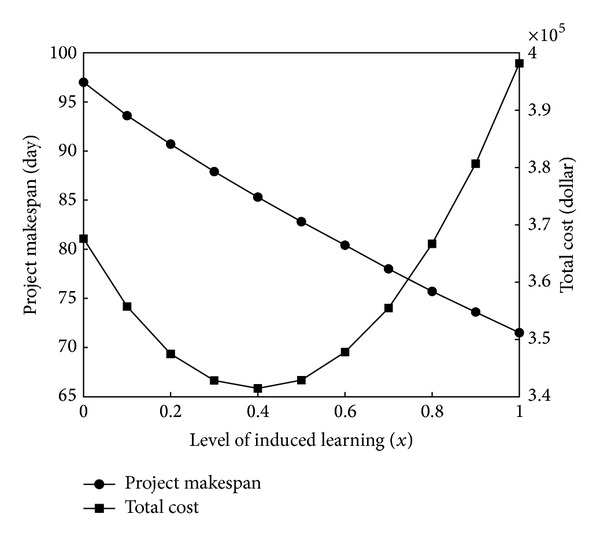
Project makespan-induced learning and total cost-induced learning curves.

Furthermore, we define *x* as the induced learning level of the four skills (**s**_1_–**s**_4_). Then, we discuss the changes in project makespan and total cost with *x*. The corresponding two curves are shown in Figure 2. It can be seen that the project makespan monotonically decreases when *x* increases from 0 to 0.1. This is because the induced learning can help the workers improve their efficiency throughout the project.

On the contrary, the total cost firstly decreases and then increases with increasing of *x*. This is because the induced learning can not only reduce cost by shortening the durations but also incur extra cost. When the reduced cost exceeds the incurred, the total cost decreases. Otherwise, the total cost increases.

The results illustrate that each level of induced learning corresponds to a combination of project makespan and total cost. By means of determining the level of induced learning, the project manager can balance them in project scheduling.

## 5. Conclusion

In this paper, we proposed a model for project scheduling problem with multiskill learning effect. In this model, both autonomous and induced learning are considered. Moreover, the learning function is sum-of-process time based, which is a suitable hypothesis for human learning. In order to solve the model, a genetic algorithm is introduced. The individuals in the algorithm are encoded in the form of activity list and decoded with SGS (serial generation scheme), maximum flow algorithm, and EFT (early finish time) priority rule. Partially matched crossover and swap mutation are applied to make the individuals feasible.

A numerical example is given to illustrate the model. The computational results show that learning effect cannot be neglected in project scheduling. The induced learning can affect both project makespan and total cost. By means of determining the level of induced learning, the project manager can balance them in project scheduling. Due to the uncertainty nature of project, the future research will extend the problem to uncertainty projects environment.

## Figures and Tables

**Table 1:** Activities of example project.

Activity no.	Successors	Normal duration (day)	Skill requirement			
			**s**_1_	**s**_2_	**s**_3_	**s**_4_
1	2, 3, 4	0	0	0	0	0
2	11	22	0	2	0	2
3	5, 6, 7	25	2	0	0	3
4	9, 10, 11	9	1	0	3	0
5	8	19	0	1	4	0
6	9, 10	13	0	4	0	3
7	9	28	0	1	0	2
8	10	3	0	3	4	0
9	12	19	2	0	0	3
10	12	13	0	4	0	2
11	12	25	0	4	0	1
12	—	0	0	0	0	0

**Table 2:** Worker-skill mapping relationships.

Worker	Skill			
	**s**_1_	**s**_2_	**s**_3_	**s**_4_
**w**_1_	1	1	1	1
**w**_2_	1	0	1	1
**w**_3_	1	1	1	0
**w**_4_	0	1	1	1
**w**_5_	1	1	0	1
**w**_6_	1	0	1	0
**w**_7_	0	1	1	0
**w**_8_	0	1	0	1
**w**_9_	1	0	0	1
**w**_10_	0	0	1	1

**Table 3:** Learning parameters on skills.

Skill	Autonomous learning ratio	Top level of induced learning	Minimum working time per unit of job (day)
**s**_1_	0.96	0.1	0.43
**s**_2_	0.95	0.1	0.41
**s**_3_	0.93	0.1	0.37
**s**_4_	0.98	0.1	0.47

**Table 4:** Optimal schedule with top level of induced learning.

Activity no.	Start time (day)	Finish time (day)	Resource allocation									
			**w**_1_	**w**_2_	**w**_3_	**w**_4_	**w**_5_	**w**_6_	**w**_7_	**w**_8_	**w**_9_	**w**_10_
1	0	0	—	—	—	—	—	—	—	—	—	—
2	0	15.8	—	—	—	—	—	—	**s**_2_	**s**_2_	**s**_4_	**s**_4_
3	0	17.6	**s**_4_	**s**_4_	**s**_1_	**s**_4_	**s**_1_	—	—	—	—	—
4	15.8	23	—	—	—	—	—	**s**_3_	**s**_3_		**s**_1_	**s**_3_
5	17.6	30.7	**s**_3_	**s**_3_	**s**_3_	**s**_3_	**s**_2_	—	—	—	—	—
6	33.6	43.2	**s**_4_	**s**_4_	**s**_2_	**s**_4_	**s**_2_	—	**s**_2_	**s**_2_	—	—
7	43.2	58.1	**s**_4_	**s**_4_	**s**_2_	—	—	—	—	—	—	—
8	30.7	33.6	**s**_3_	**s**_3_	**s**_3_	**s**_2_	**s**_2_	**s**_3_	**s**_2_	—	—	—
9	58.1	71.5	**s**_4_	**s**_4_	**s**_1_	—	—	**s**_1_	—	—	—	**s**_4_
10	43.2	51.7	—	—	—	**s**_2_	**s**_2_	—	**s**_2_	**s**_2_	**s**_4_	**s**_4_
11	51.7	65.2	—	—	—	**s**_2_	**s**_2_	—	**s**_2_	**s**_2_	**s**_4_	—
12	71.5	71.5	—	—	—	—	—	—	—	—	—	—

**Table 5:** Details on working time with top level of induced learning.

Worker	Working time in each activity (day)											
	1	2	3	4	5	6	7	8	9	10	11	12
**w**_1_	0	0	17.6	0	12.6	7.5	14.9	1.6	9.6	0	0	0
**w**_2_	0	0	17.6	0	12.6	7.5	14.9	1.6	9.6	0	0	0
**w**_3_	0	0	16.8	0	12.6	9.6	14.9	1.6	10	0	0	0
**w**_4_	0	0	17.6	0	12.6	7.5	0	2.9	0	8.5	13.1	0
**w**_5_	0	0	16.8	0	13.1	6.8	0	1.7	0	6.3	11.3	0
**w**_6_	0	0	0	5.9	0	0	0	2.8	13.4	0	0	0
**w**_7_	0	14.8	0	6.9	0	6.7	0	1.6	0	6.2	11.2	0
**w**_8_	0	14.8	0	0	0	6.8	0	0	0	6.3	11.3	0
**w**_9_	0	15.8	0	7.2	0	0	0	0	0	7.6	13.5	0
**w**_10_	0	15.8	0	6.9	0	0	0	0	10.4	7.6	0	0

**Table 6:** Optimal schedule considering no learning effect.

Activity no.	Start time (day)	Finish time (day)	Resource allocation									
			**w**_1_	**w**_2_	**w**_3_	**w**_4_	**w**_5_	**w**_6_	**w**_7_	**w**_8_	**w**_9_	**w**_10_
1	0	0	—	—	—	—	—	—	—	—	—	—
2	0	22	**s**_4_	**s**_4_	**s**_2_	**s**_2_	—	—	—	—	—	—
3	9	34	—	—	—	—	**s**_4_	**s**_1_	—	**s**_4_	**s**_1_	**s**_4_
4	0	9	—	—	—	—	**s**_1_	**s**_3_	**s**_ 3_	—	—	**s**_ 3_
5	47	66	**s**_3_	**s**_3_	**s**_3_	**s**_2_	—	**s**_3_	—	—	—	—
6	66	79	**s**_4_	**s**_4_	**s**_2_	**s**_4_	**s**_2_	—	**s**_2_	**s**_2_	—	—
7	34	62	—	—	—	—	**s**_4_	—	—	**s**_2_	**s**_4_	—
8	79	82	**s**_3_	**s**_3_	**s**_3_	**s**_ 2_	**s**_2_	**s**_3_	**s**_2_	—	—	—
9	82	101	**s**_4_	**s**_4_	**s**_1_	**s**_4_	**s**_1_	—	—	—	—	—
10	101	114	**s**_4_	**s**_4_	**s**_2_	**s**_2_	**s**_2_	—	**s**_2_	—	—	—
11	22	47	**s**_2_	**s**_4_	**s**_2_	**s**_2_	—	—	**s**_2_	—	—	—
12	114	114	—	—	—	—	—	—	—	—	—	—
